# Supplementary material for: Effects of rivastigmine on gait in patients with neurodegenerative disorders: A systematic review and meta-analysis
Source: PLoS One. 2024 Dec 12;19(12):e0310900. doi: 10.1371/journal.pone.0310900 (PMC11637393; doi:10.1371/journal.pone.0310900)
Supplement: S2 Table — (PDF) [file pone.0310900.s002.pdf]

Supporting Information

S2 Table. Search queries.

PubMed and Cochrane Library

1. Disease; colonic polyp
("Parkinson Disease"[Mesh] OR "Parkinson's Disease"[tiab] OR "Parkinson Disease"[tiab] OR "dementia"[Mesh] OR "dementia"[tiab] OR "Lewy Bodies"[Mesh] OR "Lewy Body"[tiab])

2. Intervention
("Rivastigmine"[Mesh] OR "Rivastigmine*"[tiab] OR "Exelon"[tiab] OR "ENA 713*"[tiab]) AND ("Gait"[Mesh] OR "Gait"[tiab] OR "walking"[tiab])

Embase

1. Disease; colonic polyp
('parkinson disease'/exp OR 'parkinson disease':ti,ab OR 'dementia'/exp OR 'dementia':ti,ab OR 'diffuse Lewy body disease'/exp OR 'diffuse Lewy body disease':ti,ab OR 'Lewy body disease*':ti,ab)

2. Intervention
('rivastigmine'/exp OR 'rivastigmine*':ti,ab) AND ('walking'/exp OR 'gait'/exp OR 'walking':ti,ab OR 'gait':ti,ab)

Scopus

1. Disease; colonic polyp
TITLE-ABS-KEY(Parkinson Disease)

2. Intervention
TITLE-ABS-KEY(Rivastigmine) AND (TITLE-ABS-KEY(Gait)
