# Supplementary material for: Effects of rivastigmine on gait in patients with neurodegenerative disorders: A systematic review and meta-analysis
Source: PLoS One. 2024 Dec 12;19(12):e0310900. doi: 10.1371/journal.pone.0310900 (PMC11637393; doi:10.1371/journal.pone.0310900)
Supplement: S3 Table — (PDF) [file pone.0310900.s003.pdf]

No.	Excluded Studies (screening 1)	Reason for exclusion
1	Djaldetti R, Melamed E. New drugs in the future treatment of Parkinson's disease. Journal of Neurology, Supplement. 2002;249(2):II30-II5.	unrealted topic (by title)
2	Román GC, Erkinjuntti T, Wallin A, Pantoni L, Chui HC. Subcortical ischaemic vascular dementia. Lancet Neurology. 2002;1(7):426-36. doi: 10.1016/S1474-4422(02)00190-4.	unrealted topic (by title)
3	Bäzner H, Daffertshofer M, Hennerici M. Subcortical vascular encephalopathy. Aktuelle Neurologie. 2003;30(6):266-80.	unrealted topic (by title)
4	Rascol O, Payoux P, Ory F, Ferreira JJ, Brefel-Courbon C, Montastruc JL, et al. Limitations of current Parkinson's disease therapy. Annals of Neurology. 2003;53(SUPPL. 3):S3-S15. doi: 10.1002/ana.10513.	unrealted topic (by title)
5	Román GC. Vascular dementia: Changing the paradigm. Current Opinion in Psychiatry. 2003;16(6):635-41.	unrealted topic (by title)
6	Tardieu S, Becker H, Micallef J, Blin O. Treatment of non-dopamine-dependent signs in parkinsonian syndromes: Evaluation and results. Revue Neurologique. 2003;159(5 II):3S87-3S92.	unrealted topic (by title)
7	Tariot PN. Medical management of advanced dementia. Journal of the American Geriatrics Society. 2003;51(5 SUPPL. II):S305-S13. doi: 10.1046/j.1532-5415.5156.x.	unrealted topic (by title)
8	Johnson RT, Gonzalez RG, Frosch MP. Case 27-2005: An 80-year-old man with fatigue, unsteady gait, and confusion. New England Journal of Medicine. 2005;353(10):1042-50. doi: 10.1056/NEJMcpc059024.	unrealted topic (by title)
9	Boeve BF. A review of the non-Alzheimer dementias. Journal of Clinical Psychiatry. 2006;67(12):1985-2001. doi: 10.4088/JCP.v67n1221.	unrealted topic (by title)
10	Hardesty JL. Drug therapy for dementia - Affecting more than just the mind. Topics in Geriatric Rehabilitation. 2006;22(3):243-55. doi: 10.1097/00013614-200607000-00006.	unrealted topic (by title)
11	Ramos MG, Rocha FL. Efficacy and safety of atypical antipsychotics in dementia: A sistematic review. Jornal Brasileiro de Psiquiatria. 2006;55(3):218-24. doi: 10.1590/s0047-20852006000300008.	unrealted topic (by title)
12	Cholinesterase inhibitors: Tremor and exacerbation of Parkinson's disease. Prescrire International. 2007;16(91):197-8.	unrealted topic (by title)
13	Barbui C, Cipriani A, Lintas C, Bertelé V, Garattini S. CNS drugs approved by the centralised European procedure: True innovation or dangerous stagnation? Psychopharmacology. 2007;190(2):265-8. doi: 10.1007/s00213-006-0629-3.	unrealted topic (by title)
14	Feil DG, MacLean C, Sultzer D. Quality indicators for the care of dementia in vulnerable elders. Journal of the American Geriatrics Society. 2007;55(SUPPL. 2):S293-S301. doi: 10.1111/j.1532-5415.2007.01335.x.	unrealted topic (by title)
15	Gupta P, Behari M. Akinetic rigid syndrome: An overview. Annals of Indian Academy of Neurology. 2007;10(1):21-30. doi: 10.4103/0972-2327.31481.	unrealted topic (by title)
16	Mariani E, Monastero R, Mecocci P. Mild cognitive impairment: A systematic review. Journal of Alzheimer's Disease. 2007;12(1):23-35. doi: 10.3233/JAD-2007-12104.	unrealted topic (by title)
17	Scherder EJA, Eggermont L, Sergeant J, Boersma F. Physical activity and cognition in Alzheimer's disease: Relationship to vascular risk factors, executive functions and gait. Reviews in the Neurosciences. 2007;18(2):149-58. doi: 10.1515/REVNEURO.2007.18.2.149.	unrealted topic (by title)
18	Tampi RR, van Dyck CH. Memantine: Efficacy and safety in mild-to-severe Alzheimer's disease. Neuropsychiatric Disease and Treatment. 2007;3(2):245-58. doi: 10.2147/nedt.2007.3.2.245.	unrealted topic (by title)
19	Assal F, Allali G, Kressig RW, Herrmann FR, Beauchet O. Galantamine improves gait performance in patients with Alzheimer's disease. Journal of the American Geriatrics Society. 2008;56(5):946-7. doi: 10.1111/j.1532-5415.2008.01657.x.	unrealted topic (by title)
20	Haworth JM. Gait, aging and dementia. Reviews in Clinical Gerontology. 2008;18(1):39-52. doi: 10.1017/S0959259808002700.	unrealted topic (by title)
21	Kidd PM. Alzheimer's disease, amnestic mild cognitive impairment, and age-associated memory impairment: Current understanding and progress toward integrative prevention. Alternative Medicine Review. 2008;13(2):85-115.	unrealted topic (by title)
22	Nct. Study of Rivastigmine to Treat Parkinsonian Apathy Without Dementia. https://clinicaltrialsgov/show/NCT00767091. 2008. PubMed PMID: CN-02019596.	unrealted topic (by title)

23	Staekenborg SS, van der Flier WM, van Straaten EC, Lane R, Barkhof F, Scheltens P. Neurological signs in relation to type of cerebrovascular disease in vascular dementia. Stroke. 2008;39(2):317-22. doi: 10.1161/STROKEAHA.107.493353. PubMed PMID: CN-00621914.	unrealted topic (by title)
24	Buhmann C. Therapy of geriatric patients with Parkinson's disease. Aktuelle Neurologie, Supplement. 2009;36(SUPPL. 4):S312-S9. doi: 10.1055/s-0029-1220463.	unrealted topic (by title)
25	Carecchio M, Fenoglio C, De Riz M, Guidi I, Comi C, Cortini F, et al. Progranulin plasma levels as potential biomarker for the identification of GRN deletion carriers. A case with atypical onset as clinical amnestic Mild Cognitive Impairment converted to Alzheimer's disease. Journal of the Neurological Sciences. 2009;287(1-2):291-3. doi: 10.1016/j.jns.2009.07.011.	unrealted topic (by title)
26	Dobson R, Lindahl AJ. A case of neurosyphilis mimicking dementia with Lewy bodies. Journal of Neurology, Neurosurgery and Psychiatry. 2009;80(4):461. doi: 10.1136/jnnp.2008.167387.	unrealted topic (by title)
27	Montero-Odasso M, Wells JL, Borrie MJ, Speechley M. Can cognitive enhancers reduce the risk of falls in older people with Mild Cognitive Impairment? A protocol for a randomised controlled double blind trial. 2009. p. 42.	review,abstract,conference or editoria
28	Pinkston JB, Alekseeva N, Toledo EG. Stroke and dementia. Neurological Research. 2009;31(8):824-31. doi: 10.1179/016164109X12445505689643.	unrealted topic (by title)
29	Aarsland D. Cognitive dysfunction in PD. Movement Disorders. 2010;25:S587-S8. doi: 10.1002/mds.23386.	unrealted topic (by title)
30	Aurora RN, Zak RS, Maganti RK, Auerbach SH, Casey KR, Chowdhuri S, et al. Best practice guide for the treatment of REM sleep behavior disorder (RBD). Journal of Clinical Sleep Medicine. 2010;6(1):85-95. doi: 10.5664/jcsm.27717.	unrealted topic (by title)
31	Burkhard PR. Therapeutic strategies in advanced Parkinson's disease. Schweizer Archiv fur Neurologie und Psychiatrie. 2010;161(1):33-7. doi: 10.4414/sanp.2010.02128.	unrealted topic (by title)
32	Capelli LP, Gonçalves MRR, Leite CC, Barbosa ER, Nitrini R, Vianna-Morgante AM. The fragile x-associated tremor and ataxia syndrome (FXTAS). Arquivos de Neuro-Psiquiatria. 2010;68(5):791-8. doi: 10.1590/S0004-282X201000050002	unrealted topic (by title)
33	Coelho M, Marti MJ, Tolosa E, Ferreira JJ, Valldeoriola F, Rosa M, et al. Late-stage Parkinson's disease: The Barcelona and Lisbon cohort. Journal of Neurology. 2010;257(9):1524-32. doi: 10.1007/s00415-010-5566-8.	unrealted topic (by title)
34	Nct. Effects of Exercise and Rivastigmine on Quality of Life of Alzheimer's Disease Patients. https://clinicaltrialsgov/show/NCT01183806. 2010. PubMed PMID: CN-02019402.	unrealted topic (by title)
35	Sazci A, Idrisoglu HA. Neurodegenerative disorders' epidemiology, clinics, genetics, pharmacogenetics and imaging. Clinical Genetics. 2010;78:1-3. doi: 10.1111/j.1399-0004.2010.01583.x.	unrealted topic (by title)
36	Tucker I. Management of inappropriate sexual behaviors in dementia: A literature review. International Psychogeriatrics. 2010;22(5):683-92. doi: 10.1017/S1041610210000189.	unrealted topic (by title)
37	Grigg-Damberger M, Ralls F. Primary sleep disorders and paroxysmal nocturnal nonepileptic events in adults with epilepsy from the perspective of sleep specialists. Journal of Clinical Neurophysiology. 2011;28(2):120-40. doi: 10.1097/WNP.0b013e3182120fed.	unrealted topic (by title)
38	Sivakumar V, Anand S. Functional imaging of the nigro-striatal pathway: A clinical review. Indian Journal of Psychiatry. 2011;53(5):S29.	unrealted topic (by title)
39	Xiong YY, Mok V. Age-related white matter changes. Journal of Aging Research. 2011;2011. doi: 10.4061/2011/617927.	unrealted topic (by title)
40	Contreras A, Grandas F. Risk factors for freezing of gait in Parkinson's disease. Journal of the Neurological Sciences. 2012;320(1-2):66-71. doi: 10.1016/j.jns.2012.06.018.	unrealted topic (by title)
41	Lauretani F, Maggio M, Silvestrini C, Nardelli A, Saccavini M, Ceda GP. Parkinson's disease (PD) in the elderly: An example of geriatric syndrome (GS)? Archives of Gerontology and Geriatrics. 2012;54(1):242-6. doi: 10.1016/j.archger.2011.03.002.	unrealted topic (by title)
42	Lott IT. Neurological phenotypes for Down syndrome across the life span. 2012. p. 101-21.	unrealted topic (by title)
43	Velentzas I, Seferis H, Afentouli P, Torrens M, Tagaris G. Two years observation after pedunculopontine nucleus (PPN) DBS surgery of two female patients with progressive supranuclear palsy (PSP). Movement Disorders. 2012;27:S324-S5. doi: 10.1002/mds.25051.	unrealted topic (by title)

44	Xiaolu C. Management of dementia,a nonmotor feature of Parkinson disease. Molecular Neurobiology. 2012;46:S39. doi: 10.1007/s12035-012-8319-0.	unrealted topic (by title)
45	Bugalho P, Viana-Baptista M. REM sleep behavior disorder and motor dysfunction in Parkinson's disease - A longitudinal study. Parkinsonism and Related Disorders. 2013;19(12):1084-7. doi: 10.1016/j.parkreldis.2013.07.017.	unrealted topic (by title)
46	Chua IC, Sendoya S, Perez F. Cerebral amyloid angiopathy (CAA). Journal of the American Geriatrics Society. 2013;61:S21. doi: 10.1111/jgs.12263.	unrealted topic (by title)
47	Devos D, Moreau C, Dujardin K, Cabantchik I, Defebvre L, Bordet R. New pharmacological options for treating advanced parkinson's disease. Clinical Therapeutics. 2013;35(10):1640-52. doi: 10.1016/j.clinthera.2013.08.011.	unrealted topic (by title)
48	Golbe LI. Diagnosis and management of progressive supranuclear palsy. Neurodegenerative Disease Management. 2013;3(1):81-90. doi: 10.2217/nmt.13.1.	unrealted topic (by title)
49	Hilaly SMR, Chowdhury MTI, Chowdhury MSJH. Trends in managing Parkinson's disease - A review. Journal of Medicine (Bangladesh). 2013;14(2):174-84. doi: 10.3329/jom.v14i2.19670.	unrealted topic (by title)
50	Isik AT, Soysal P, Comert B, Mas MR. Effects of cholinesterase inhibitors on gait and balance in elderly people with Alzheimer's disease. Alzheimer's and Dementia. 2013;9(4):P654. doi: 10.1016/j.jalz.2013.05.1340.	unrealted topic (by title)
51	Kalia LV, Brotchie JM, Fox SH. Novel nondopaminergic targets for motor features of Parkinson's disease: Review of recent trials. Movement Disorders. 2013;28(2):131-44. doi: 10.1002/mds.25273.	unrealted topic (by title)
52	Tan SB, Williams A. The effects of Parkinson's disease on caregivers and people with Parkinson's disease: A literature review. Proceedings of Singapore Healthcare. 2013;22(3):191-7. doi: 10.1177/201010581302200306.	unrealted topic (by title)
53	Hanci E, Balci-Sengul MC, Sendur I, Civlan S. A case report: Normal pressure hydrocephalus diagnosis after being followed as dementia. Klinik Psikofarmakoloji Bulteni. 2014;24:S102.	unrealted topic (by title)
54	Madhusoodanan S, Wilkes V, Campbell RP, Serper M, Essuman EK, Brenner R. Psychiatric symptoms of progressive supranuclear palsy: A case report and brief review. Neuropsychiatry. 2014;4(1):27-32. doi: 10.2217/npy.13.81.	unrealted topic (by title)
55	Sarva H, Deik A, Swan MC, Severt WL. Freezing of gait after a thalamic hemorrhage can respond to venlafaxine and rivastigmine. Movement Disorders. 2014;29:S309. doi: 10.1002/mds.25914.	unrealted topic (by title)
56	Ransmayr G. Cognitive impairment in Parkinson's disease. Psychiatria Danubina. 2015;27(4):458-61.	unrealted topic (by title)
57	Reichmann H. Synucleinopathies (PDD-LBD-MSA). European Journal of Neurology. 2015;22:860. doi: 10.1111/ene.12813.	unrealted topic (by title)
58	Sommer M, Stiksrud EM, von Eckardstein K, Rohde V, Paulus W. When battery exhaustion lets the lame walk: A case report on the importance of long-term stimulator monitoring in deep brain stimulation. BMC Neurology. 2015;15(1). doi: 10.1186/s12883-015-0365-6.	unrealted topic (by title)
59	Abbate C, Caputo L, Damanti S, Zappa C, Nicolini P, Rossi PD, et al. Reversible Parkinson's Dementia Associated with Withdrawal of Androgen-Deprivation Therapy for Prostate Cancer. Journal of the American Geriatrics Society. 2016;64(10):e115-e7. doi: 10.1111/jgs.14417.	unrealted topic (by title)
60	Alvira Rasal B, Martín De Francisco E. Rapidly progressive dementia; a case of corticobasal degeneration with atypical presentation. European Geriatric Medicine. 2016;7:S51.	unrealted topic (by title)
61	Amadori K. Rivastigmine and Parkinson's disease without dementia : Impact on gait stability and frequency of falling. Zeitschrift fur Gerontologie und Geriatrie. 2016;49(7):662-3.	review,abstract,conference or editoria
62	Lord SR. Virtual reality and the prevention of falls in the real world. The Lancet. 2016;388(10050):1132-4. doi: 10.1016/S0140-6736(16)31347-2.	unrealted topic (by title)
63	Nct. Efficacy of RIVAstigmine on Motor, Cognitive and Behavioural Impairment in Progressive Supranuclear Palsy. https://clinicaltrialsgov/show/NCT02839642. 2016. PubMed PMID: CN-01582897.	unrealted topic (by title)
64	Perez-Lloret S, Peralta MC, Barrantes FJ. Pharmacotherapies for Parkinson’s disease symptoms related to cholinergic degeneration. Expert Opinion on Pharmacotherapy. 2016;17(18):2405-15. doi: 10.1080/14656566.2016.1254189.	unrealted topic (by title)

65	Zhao Y, Nonnekes J, Storcken EJM, Janssen S, van Wegen EEH, Bloem BR, et al. Feasibility of external rhythmic cueing with the Google Glass for improving gait in people with Parkinson’s disease. Journal of Neurology. 2016;263(6):1156-65. doi: 10.1007/s00415-016-8115-2.	unrealted topic (by title)
66	Bhattacharjee S, Malone D, Warholak T, Knapp S, Lo-Ciganic W, Lee J, et al. Comparison of fall and fracture risk for medications to treat Alzheimer's disease. Journal of Managed Care and Specialty Pharmacy. 2017;23:S51-S2.	unrealted topic (by title)
67	Castellano CA, Paquet N, Dionne IJ, Imbeault H, Langlois F, Croteau E, et al. A 3-Month Aerobic Training Program Improves Brain Energy Metabolism in Mild Alzheimer's Disease: Preliminary Results from a Neuroimaging Study. Journal of Alzheimer's Disease. 2017;56(4):1459-68. doi: 10.3233/JAD-161163.	unrealted topic (by title)
68	Henderson EJ, Smith N, Gaunt DM, Lawrence AD, Brodie MA, Close JCT, et al. Does the anticholinergic burden of drugs predict outcomes in people with parkinson's disease with a history of a fall? Age and Ageing. 2017;46:i44. doi: 10.1093/ageing/afx062.158.	review,abstract,conference or editoria
69	Umin. Investigation of the effect of rivastigmine patch on gait disturbance in patients with Alzheimer disease. Clinical study (intervention study). https://trialsearchwhoint/Trial2aspx?TrialID=JPRN-UMIN000025869. 2017. PubMed PMID: CN-01824669.	review,abstract,conference or editoria
70	Actrn. Maintain Your Brain (MYB): a 3-year study of a personalised online program to prevent cognitive decline and incident dementia amongst community dwelling 55-77 year olds. https://trialsearchwhoint/Trial2aspx?TrialID=ACTRN12618000851268. 2018. PubMed PMID: CN-01900320.	unrealted topic (by title)
71	Alsomali H, O'Mara G. A curious case of Parkinsonism. Irish Journal of Medical Science. 2018;187(3):S34-S5. doi: 10.1007/s11845-018-1833-y.	unrealted topic (by title)
72	Fox SH, Katzenschlager R, Lim SY, Barton B, de Bie RMA, Seppi K, et al. International Parkinson and movement disorder society evidence-based medicine review: Update on treatments for the motor symptoms of Parkinson's disease. Movement Disorders. 2018;33(8):1248-66. doi: 10.1002/mds.27372.	unrealted topic (by title)
73	Gilat M, Lígia Silva de Lima A, Bloem BR, Shine JM, Nonnekes J, Lewis SJG. Freezing of gait: Promising avenues for future treatment. Parkinsonism and Related Disorders. 2018;52:7-16. doi: 10.1016/j.parkreldis.2018.03.009.	unrealted topic (by title)
74	Hamada K, Kishimoto RI, Yuasa T. Triple neurotransmitter replacement therapy can improve symptoms in PSP patients. Clinical Neurology. 2018;58:S399. doi: 10.5692/clinicalneurol.58-supplement-S250.	unrealted topic (by title)
75	Kılıç Ş, Yılmaz NH, Hanoğlu L, Özer FF. Lower extremity tremor exacerbation due to rivastigmine administration for treatment of Parkinson’s Disease Dementia. Gazi Medical Journal. 2018;29(2):134-5. doi: 10.12996/gmj.2018.36.	unrealted topic (by title)
76	Lamb SE, Sheehan B, Atherton N, Nichols V, Collins H, Mistry D, et al. Dementia And Physical Activity (DAPA) trial of moderate to high intensity exercise training for people with dementia: Randomised controlled trial. BMJ (Online). 2018;361. doi: 10.1136/bmj.k1675.	unrealted topic (by title)
77	Lord SR, Close JCT. New horizons in falls prevention. Age and Ageing. 2018;47(4):492-8. doi: 10.1093/ageing/afy059.	unrealted topic (by title)
78	Matsuura K, Kajikawa H, Tabei KI, Satoh M, Kida H, Nakamura N, et al. The effectiveness of istradefylline for the treatment of gait deficits and sleepiness in patients with Parkinson's disease. Neuroscience Letters. 2018;662:158-61. doi: 10.1016/j.neulet.2017.10.018.	unrealted topic (by title)
79	Smith MD, Peall KJ. Repurposed drugs for use in Parkinson’s disease. Journal of Neurology. 2018;265(3):728-30. doi: 10.1007/s00415-018-8772-4.	unrealted topic (by title)
80	Amadori K, Steiner T. Parkinson’s syndromes in geriatric patients: Epidemiological, clinical and therapeutic characteristics. Nervenarzt. 2019;90(12):1279-91. doi: 10.1007/s00115-019-00825-8.	unrealted topic (by title)
81	Arvanitakis Z, Shah RC, Bennett DA. Diagnosis and Management of Dementia: Review. JAMA - Journal of the American Medical Association. 2019;322(16):1589-99. doi: 10.1001/jama.2019.4782.	unrealted topic (by title)

82	Ates Bulut E, Karabay N, Soysal P, Isik AT. An elderly patient with Alzheimer’s disease, normal pressure hydrocephalus and traumatic brain injury: presented with behavioral symptoms similar to behavioral variant frontotemporal dementia. International Journal of Neuroscience. 2019;129(6):623-6. doi: 10.1080/00207454.2018.1552690.	unrealted topic (by title)
83	Caviness JN. Treatment of myoclonus in degenerative disorders. 2019. p. 277-80.	unrealted topic (by title)
84	Isrctn. A trial of rivastigmine to prevent falls in Parkinson's. https://trialsearchwhoint/Trial2aspx?TrialID=ISRCTN41639809. 2019. PubMed PMID: CN-01972311.	review,abstract,conference or editoria
85	Morris R, Martini DN, Madhyastha T, Kelly VE, Grabowski TJ, Nutt J, et al. Overview of the cholinergic contribution to gait, balance and falls in Parkinson's disease. Parkinsonism and Related Disorders. 2019;63:20-30. doi: 10.1016/j.parkreldis.2019.02.017.	review,abstract,conference or editoria
86	Nguyen J, Papesh K. Potential treatment considerations for refractory REM behavior disorder. Movement Disorder. 2019;34:S162.	unrealted topic (by title)
87	Pirker W. E.07.02 New strategies for therapy throughout the different stages of Parkinson's disease. European Neuropsychopharmacology. 2019;29:S34. doi: 10.1016/j.euroneuro.2018.11.1101.	unrealted topic (by title)
88	Saeed S, Jeong D, Mohammad A, Cong L. Syncope secondary to rivastigmine use in older adults. Journal of the American Geriatrics Society. 2019;67:S278. doi: 10.1111/jgs.15898.	unrealted topic (by title)
89	Abay RJ, Gold LS, Andrews J. Sarcopenia and ADL disability among hospitalized older adults with dementia in the health ABC study. Journal of the American Geriatrics Society. 2020;68(SUPPL 1):S297. doi: 10.1111/jgs.16431.	unrealted topic (by title)
90	Gao C, Liu J, Tan Y, Chen S. Freezing of gait in Parkinson's disease: Pathophysiology, risk factors and treatments. Translational Neurodegeneration. 2020;9(1). doi: 10.1186/s40035-020-00191-5.	unrealted topic (by title)
91	Gonzalez-Latapi P, Bhowmick SS, Saranza G, Fox SH. Non-Dopaminergic Treatments for Motor Control in Parkinson’s Disease: An Update. CNS Drugs. 2020;34(10):1025-44. doi: 10.1007/s40263-020-00754-0.	unrealted topic (by title)
92	Kang DW, Wang SM, Um YH, Na HR, Kim NY, Han K, et al. Differential Risk of Incident Fractures Depending on Intensity and Frequency of Physical Activity According to Cognitive Status: A Nationwide Longitudinal Study. Frontiers in Medicine. 2020;7. doi: 10.3389/fmed.2020.572466.	unrealted topic (by title)
93	Nct. TRANSCRANIAL DIRECT CURRENT STIMULATION (t-DCS) AS ADD-ON TO NEUROREHABILITATION OF PISA SYNDROME IN PARKINSON DISEASE. https://clinicaltrialsgov/show/NCT04620863. 2020. PubMed PMID: CN-02197358.	unrealted topic (by title)
94	Grobe-Einsler M, Vogt IR, Schaprian T, Hurlemann R, Klockgether T, Kaut O. Effects of Rivastigmine on Patients with Spinocerebellar Ataxia Type 3: A Case Series of Five Patients. Neurodegenerative Diseases. 2021;20(2-3):104-9. doi: 10.1159/000510057.	unrealted topic (by title)
95	Megherbi L, Si Ahmed H, Daoudi S. Unusual progressive supranuclear Palsy: A case report. Movement Disorder. 2021;36(SUPPL 1):S252. doi: 10.1002/mds.28794.	unrealted topic (by title)
96	Pinto M, Castro Caldas A, Guimaraes J, Massano J, Ferreira J, Soares Dos Reis R. Seronegative autoimmune encephalitis presenting as a rapidly-progressive cognitive and movement disorder. European Journal of Neurology. 2021;28(SUPPL 1):853. doi: 10.1111/ene.14975.	unrealted topic (by title)
97	Todorov I, Kostov K. Review on electroencephalographic findings in patients with alzheimer’s disease. Journal of IMAB - Annual Proceeding (Scientific Papers). 2021;27(1):3529-33. doi: 10.5272/jimab.2021271.3529.	unrealted topic (by title)
98	Belo T, Batista Correia J, Pereira I, Fernandes P, Argel M, Ferro R, et al. Amyotrophic lateral sclerosis meets frontotemporal dementia - A case report. Journal of Sleep Research. 2022;31. doi: 10.1111/jsr.13740.	unrealted topic (by title)
99	Dhavala A, Samitinjay A, Khairkar P, Podder V, Price A, Fatima SH, et al. Integrated case-based clinical approach in understanding pathways, complexities, pitfalls and challenges in neurodegenerative disorders. American Journal of Neurodegenerative Diseases. 2022;11(2):22-33.	unrealted topic (by title)
100	Holter ST, Nonnekes J, Bloem B. Music to move persons with Parkinson’s disease: a personalized approach. Journal of Neurology. 2022;269(1):251-2. doi: 10.1007/s00415-021-10615-5.	unrealted topic (by title)

101	Kartanou C, Kontogeorgiou Z, Rentzos M, Potagas C, Aristeidou S, Kapaki E, et al. Expanding the spectrum of C9ORF72-related neurodegenerative disorders in the Greek population. Journal of the Neurological Sciences. 2022;442. doi: 10.1016/j.jns.2022.120450.	unrealted topic (by title)
102	Luque-Buzo E, Pérez Sáchez J, Gonzalez-Sanchez M, Contreras-Chicote A, Secades S, Casa-Fages B, et al. Asymmetric Early Onset Parkinsonism Due To PSEN1 Mutation. Movement Disorders. 2022;37:S290. doi: 10.1002/mds.29223.	unrealted topic (by title)
103	Pérez Palmer N, Trejo Ortega B, Joshi P. Cognitive Impairment in Older Adults: Epidemiology, Diagnosis, and Treatment. Psychiatric Clinics of North America. 2022;45(4):639-61. doi: 10.1016/j.psc.2022.07.010.	unrealted topic (by title)
104	Su C, Yang X, Wei S, Zhao R. Association of Cerebral Small Vessel Disease With Gait and Balance Disorders. Frontiers in Aging Neuroscience. 2022;14. doi: 10.3389/fnagi.2022.834496.	unrealted topic (by title)
105	Tiepolt S, Meyer PM, Patt M, Deuther-Conrad W, Hesse S, Barthel H, et al. PET Imaging of Cholinergic Neurotransmission in Neurodegenerative Disorders. Journal of Nuclear Medicine. 2022;63:33S-44S. doi: 10.2967/JNUMED.121.263198.	unrealted topic (by title)
106	Ukai K. A case of limited cutaneous systemic sclerosis with non-Fahr-type calcification in the brain and a review of the literature. Psychogeriatrics. 2022;22(6):882-5. doi: 10.1111/psyg.12887.	unrealted topic (by title)
107	Devi G. A how-to guide for a precision medicine approach to the diagnosis and treatment of Alzheimer's disease. Frontiers in Aging Neuroscience. 2023;15. doi: 10.3389/fnagi.2023.1213968.	unrealted topic (by title)
108	Elefante C, Brancati GE, Torrigiani S, Amadori S, Ricciardulli S, Pistolesi G, et al. Bipolar Disorder and Manic-Like Symptoms in Alzheimer's, Vascular and Frontotemporal Dementia: A Systematic Review. Current Neuropharmacology. 2023;21(12):2516-42. doi: 10.2174/1570159X20666220706110157.	unrealted topic (by title)
109	Ishida T, Murayama T, Kobayashi S. Current research of idiopathic normal pressure hydrocephalus: Pathogenesis, diagnosis and treatment. World Journal of Clinical Cases. 2023;11(16):3706-13. doi: 10.12998/wjcc.v11.i16.3706.	unrealted topic (by title)
110	Neylan KD, Miller BL. New Approaches to the Treatment of Frontotemporal Dementia. Neurotherapeutics. 2023;20(4):1055-65. doi: 10.1007/s13311-023-01380-6.	unrealted topic (by title)
111	Arahata H, Ohyagi Y, Matsumoto S, Furuya H, Murai H, Kuwabara Y, et al. [A patient with probable dementia with Lewy bodies, who showed improvement of dementia and parkinsonism by the administratim of donepezil]. Rinsho Shinkeigaku. 2001;41(7):402-6. Epub 2002/01/26. PubMed PMID: 11808350.	unrealted topic (by title)
112	Nnodim JO, Alexander NB. Assessing falls in older adults: A comprehensive fall evaluation to reduce fall risk in older adults. Geriatrics. 2005;60(10):24-8.	unrealted topic (by title)
113	Pal PK, Netravathi M. Management of neurodegenerative disorders: Parkinson's disease and Alzheimer's disease. J Indian Med Assoc. 2005;103(3):168-70, 72, 74-6. Epub 2005/09/22. PubMed PMID: 16173294.	unrealted topic (by title)
114	Gupta P, Behari M. Akinetic rigid syndrome: An overview. Annals of Indian Academy of Neurology. 2007;10(1):21-30. doi: 10.4103/0972-2327.31481.	unrealted topic (by title)
115	Scoyni RM, Aiello L, Trani I, Felli B, Masin AM, Camponi V, et al. Drug adverse events and drop-out risk: a clinical case. Arch Gerontol Geriatr. 2007;44 Suppl 1:359-64. Epub 2007/02/24. doi: 10.1016/j.archger.2007.01.049. PubMed PMID: 17317475.	unrealted topic (by title)
116	Litvinenko IV, Odinak MM, Mogil'naya VI, Emelin AY. Efficacy and safety of galantamine (reminyl) for dementia in patients with Parkinson's disease (an open controlled trial). Neurosci Behav Physiol. 2008;38(9):937-45. Epub 2008/11/01. doi: 10.1007/s11055-008-9077-3. PubMed PMID: 18975103.	unrealted topic (by title)
117	Nct. Donepezil and the Risk of Falls in Seniors With Cognitive Impairment. https://clinicaltrialsgov/show/NCT00934531. 2009. PubMed PMID: CN-02043166.	unrealted topic (by title)
118	Lucetti C, Logi C, Del Dotto P, Berti C, Ceravolo R, Baldacci F, et al. Levodopa response in dementia with lewy bodies: A 1-year follow-up study. Parkinsonism and Related Disorders. 2010;16(8):522-6. doi: 10.1016/j.parkreldis.2010.06.004.	unrealted topic (by title)
119	Poewe W. Current and future perspectives in the treatment of Parkinson's disease. European Journal of Neurology. 2010;17:647. doi: 10.1111/j.1468-1331.2010.03235.x.	unrealted topic (by title)

120	Adams C, Keep M, Martin K, McVicker J, Kumar R. Acute induction of levodopa-resistant freezing of gait upon subthalamic nucleus electrode implantation. Movement Disorders. 2011;26:S65. doi: 10.1002/mds.23764.	unrealted topic (by title)
121	Segev-Jacubovski O, Herman T, Yogev-Seligmann G, Mirelman A, Giladi N, Hausdorff JM. The interplay between gait, falls and cognition: Can cognitive therapy reduce fall risk? Expert Review of Neurotherapeutics. 2011;11(7):1057-75. doi: 10.1586/ern.11.69.	review,abstract,conference or editoria
122	Weintraub D, Morgan JC. Both the body and brain benefit from exercise: Potential win-win for Parkinson's disease patients. Movement Disorders. 2011;26(4):607. doi: 10.1002/mds.23726.	unrealted topic (by title)
123	Yarnall A, Rochester L, Burn DJ. The interplay of cholinergic function, attention, and falls in Parkinson's disease. Mov Disord. 2011;26(14):2496-503. Epub 2011/09/08. doi: 10.1002/mds.23932. PubMed PMID: 21898597.	unrealted topic (by title)
124	Boczarska-Jedynak M, Stompel D, Jasinska-Myga B, Flak M, Czechowicz B, Opala G. Isolated backward gait disturbances as an early sign of progressive supranuclear palsy. Case report. Movement Disorders. 2012;27:S374. doi: 10.1002/mds.25051.	unrealted topic (by title)
125	Litvinenko IV, Khalimov RR, Trufanov AG, Krasakov IV, Khaĭmov DA. [New approach to gait disorders therapy in late stages of Parkinson's disease]. Adv Gerontol. 2012;25(2):267-74. Epub 2012/11/08. PubMed PMID: 23130517.	unrealted topic (by title)
126	Nct. The Effect of Donepezil on Gait and Balance in Parkinson's Disease. https://clinicaltrialsgov/show/NCT01521117. 2012. PubMed PMID: CN-01535245.	review,abstract,conference or editoria
127	Ebersbach G, Moreau C, Gandor F, Defebvre L, Devos D. Clinical syndromes: Parkinsonian gait. Movement Disorders. 2013;28(11):1552-9. doi: 10.1002/mds.25675.	unrealted topic (by title)
128	Kim SD, Allen NE, Canning CG, Fung VS. Postural instability in patients with Parkinson's disease. Epidemiology, pathophysiology and management. CNS Drugs. 2013;27(2):97-112. Epub 2012/10/19. doi: 10.1007/s40263-012-0012-3. PubMed PMID: 23076544.	review,abstract,conference or editoria
129	Hübscher A, Isenmann S. Multi-factorial gait disorders and falls - A neuro-geriatric perspective. Nervenheilkunde. 2014;33(7-8):527-34. doi: 10.1055/s-0038-1627712.	unrealted topic (by title)
130	van den Heuvel MRC, Kwakkel G, Beek PJ, Berendse HW, Daffertshofer A, van Wegen EEH. Effects of augmented visual feedback during balance training in Parkinson's disease: A pilot randomized clinical trial. Parkinsonism and Related Disorders. 2014;20(12):1352-8. doi: 10.1016/j.parkreldis.2014.09.022.	unrealted topic (by title)

No.	Excluded Studies (screening 2)	Reason for exclusion
1	Caballol N, Martí MJ, Tolosa E. Cognitive dysfunction and dementia in Parkinson disease. Movement Disorders. 2007;22(SUPPL. 17):S358-S66. doi: 10.1002/mds.21677.	not RCT
2	Moretti R, Torre P, Antonello RM, Cazzato G, Pizzolato G. Different responses to rivastigmine in subcortical vascular dementia and multi-infarct dementia. American Journal of Alzheimer's Disease and other Dementias. 2008;23(2):167-76. doi: 10.1177/1533317507312558.	not RCT
3	Olin JT, Aarsland D, Meng X. Rivastigmine in the treatment of dementia associated with parkinson's disease (PDD): Subscale analysis of Activities of Daily Living (ADL). Annals of Neurology. 2009;66:S45. doi: 10.1002/ana.21857.	no quantified outcome
4	Devos D, Bordet R, Defebvre L. Pharmacological hypotheses and therapeutic strategies for gait disorders in Parkinson's disease. Revue Neurologique. 2010;166(2):168-77. doi: 10.1016/j.neurol.2009.07.017.	not RCT
5	Devos D, Defebvre L, Bordet R. Dopaminergic and non-dopaminergic pharmacological hypotheses for gait disorders in Parkinson's disease. Fundamental and Clinical Pharmacology. 2010;24(4):407-21. doi: 10.1111/j.1472-8206.2009.00798.x.	not RCT
6	Kim D, Brown R, Berry S. Cognitive enhancers and risk of fall-related adverse events - Systematic review and meta-analysis of randomized controlled trials. Journal of the American Geriatrics Society. 2010;58:S99. doi: 10.1111/j.1532-5415.2010.02850.x.	not RCT
7	Henderson EJ, Lord SR, Close JC, Lawrence AD, Whone A, Ben-Shlomo Y. The ReSPonD trial--rivastigmine to stabilise gait in Parkinson's disease a phase II, randomised, double blind, placebo controlled trial to evaluate the effect of rivastigmine on gait in patients with Parkinson's disease who have fallen. BMC neurology. 2013;13:188. doi: 10.1186/1471-2377-13-188. PubMed PMID: CN-00973261.	not RCT
8	Beauchet O, Launay CP, Allali G, Herrmann FR, Annweiler C. Gait changes with anti-dementia drugs: A prospective, open-label study combining single and dual task assessments in patients with Alzheimer's disease. Drugs and Aging. 2014;31(5):363-72. doi: 10.1007/s40266-014-0175-3.	retracted (include all ACE inhibitors)
9	Pagano G, Rengo G, Pasqualetti G, Femminella GD, Monzani F, Ferrara N, et al. Cholinesterase inhibitors for Parkinson's disease: A systematic review and meta-analysis. Journal of Neurology, Neurosurgery and Psychiatry. 2015;86(7):767-73. doi: 10.1136/jnnp-2014-308764.	not RCT
10	Paiva P, Molho EH, Ramirez-Zamora A. Treatment of secondary freezing of gait with rivastigmine. Movement Disorders. 2015;30:S428. doi: 10.1002/mds.26295.	not RCT
11	Beauchet O, Barden J, Liu-Ambrose T, Chester VL, Annweiler C, Szturm T, et al. Anti-Dementia Drugs, Gait Performance and Mental Imagery of Gait: A Non-Randomized Open-Label Trial. Drugs and Aging. 2016;33(9):665-73. doi: 10.1007/s40266-016-0391-0.	no quantified outcome
12	Isik AT, Soysal P, Usarel C. Effects of Acetylcholinesterase Inhibitors on Balance and Gait Functions and Orthostatic Hypotension in Elderly Patients with Alzheimer Disease. American Journal of Alzheimer's Disease and other Dementias. 2016;31(7):580-4. doi: 10.1177/1533317516666195.	no quantified outcome
13	Moreau C, Devos D, Defebvre L. Acetylcholinesterase inhibitors and gait: a steadying hand? The Lancet Neurology. 2016;15(3):232-3. doi: 10.1016/S1474-4422(16)00003-X.	not RCT
14	Smulders K, Dale ML, Carlson-Kuhta P, Nutt JG, Horak FB. Pharmacological treatment in Parkinson's disease: Effects on gait. Parkinsonism and Related Disorders. 2016;31:3-13. doi: 10.1016/j.parkreldis.2016.07.006.	not RCT
15	Taylor JL, Sumukadas D. Should ReSPonD change falls prevention in Parkinson's disease? Journal of the Royal College of Physicians of Edinburgh. 2016;46(2):101-2. doi: 10.4997/JRCPE.2016.108.	not RCT
16	Falkenburger B. ExPPNing how acetylcholine improves gait in Parkinson's disease: An Editorial Highlight for 'Deletion of the Vesicular Acetylcholine Transporter from Pedunculopontine/laterodorsal tegmental neurons modifies gait'. Journal of Neurochemistry. 2017;140(5):688-91. doi: 10.1111/jnc.13899.	not RCT
17	Debû B, De Oliveira Godeiro C, Lino JC, Moro E. Managing Gait, Balance, and Posture in Parkinson's Disease. Current Neurology and Neuroscience Reports. 2018;18(5). doi: 10.1007/s11910-018-0828-4.	not RCT
18	Koshy Cherian A, Kucinski A, Wu R, de Jong IEM, Sarter M. Co-treatment with rivastigmine and idalopirdine reduces the propensity for falls in a rat model of falls in Parkinson's disease. Psychopharmacology. 2019;236(6):1701-15. doi: 10.1007/s00213-018-5150-y.	retracted (not human study)
19	Neumann S, Taylor J, Bamford A, Metcalfe C, Gaunt DM, Whone A, et al. CHIEF-PD: Cholinesterase Inhibitor to prEvent Falls in Parkinson's Disease. Movement Disorders. 2020;35(SUPPL 1):S409. doi: 10.1002/mds.28268.	not RCT

20	Cui CK, Lewis SJG. Future Therapeutic Strategies for Freezing of Gait in Parkinson’s Disease. Frontiers in Human Neuroscience. 2021;15. doi: 10.3389/fnhum.2021.741918.	not RCT
21	Neumann S, Taylor J, Bamford A, Metcalfe C, Gaunt DM, Whone A, et al. Cholinesterase inhibitor to prevent falls in Parkinson's disease (CHIEF-PD) trial: a phase 3 randomised, double-blind placebo-controlled trial of rivastigmine to prevent falls in Parkinson's disease. BMC neurology. 2021;21(1):422. doi: 10.1186/s12883-021-02430-2. PubMed PMID: CN-02339853.	not RCT
22	Smith MD, Brazier DE, Henderson EJ. Current perspectives on the assessment and management of gait disorders in parkinson’s disease. Neuropsychiatric Disease and Treatment. 2021;17:2965-85. doi: 10.2147/NDT.S304567.	not RCT
23	McDonald J, Pourcher E, Nadeau A, Corbeil P. A Randomized Trial of Oral and Transdermal Rivastigmine for Postural Instability in Parkinson Disease Dementia. Clin Neuropharmacol. 2018;41(3):87-93. doi: 10.1097/WNF.0000000000000275. PubMed PMID: 29537978.	retracted (used different outcome parameters)

No.	Included Studies
1	Gurevich T, Balash Y, Merims D, Peretz C, Herman T, Hausdorff JM, et al. Effect of rivastigmine on mobility of patients with higher-level gait disorder: a pilot exploratory study. Drugs in R&D. 2014;14(2):57-62. doi: 10.1007/s40268-014-0038-8. PubMed PMID: CN-00999652.
2	Henderson EJ, Lord SR, Brodie MA, Gaunt DM, Lawrence AD, Close JC, et al. Rivastigmine for gait stability in patients with Parkinson's disease (ReSPonD): a randomised, double-blind, placebo-controlled, phase 2 trial. The lancet Neurology. 2016;15(3):249-58. doi: 10.1016/S1474-4422(15)00389-0. PubMed PMID: CN-01168379.
3	Shimura H, Saiko A, Hayashi A, Hattori N, Urabe T. Rivastigmine improves dual-task gait velocity in patients with Alzheimer's disease. BMC Neurology. 2021;21(1). doi: 10.1186/s12883-021-02098-8.
4	Li Z, Yu Z, Zhang J, Wang J, Sun C, Wang P, et al. Impact of Rivastigmine on Cognitive Dysfunction and Falling in Parkinson's Disease Patients. Eur Neurol. 2015;74(1-2):86-91. Epub 20150820. doi: 10.1159/000438824. PubMed PMID: 26288230.
