# Supplementary material for: Effects of rivastigmine on gait in patients with neurodegenerative disorders: A systematic review and meta-analysis
Source: PLoS One. 2024 Dec 12;19(12):e0310900. doi: 10.1371/journal.pone.0310900 (PMC11637393; doi:10.1371/journal.pone.0310900)
Supplement: S4 Table — (PDF) [file pone.0310900.s004.pdf]

Supplementary Table 4. Characteristics of the Included studies

Study	Outcome (unit)	RS_n	RS_MD_org	RS_sd_org	RS_MD_m.sec	RS_sd_m.sec	RS_se_m.sec	Pla_n	Pla_MD	Pla_sd	Hedges' g	SEg	RS_MD_std	RS_se_std	Names of data extractor	Date of data extraction
Shimura 2021	Gait speed (m/min)	21	0.61	11.85	0.01	0.20	0.04						0.05	0.22	Shim SR, Lee SM	Apr-24
Gurevich 2014	Gait speed (m/sec)	15	0.04	0.76									0.05	0.26	Shim SR, Lee SM	May-24
Henderson 2016	Gait speed (m/sec), Fall number (per month)	55	1.08	0.29	1.08	0.29	0.04						3.72	0.38	Shim SR, Lee SM	Jun-24
Henderson 2016	Gait speed (m/sec), Fall number (per month)	55			1.40	2.47	0.33	59	2.40	4.40	-0.28	0.19			Shim SR, Lee SM	Jun-24
Li 2015	Fall number (per year)	41			1.82	1.99	0.31	40	13.00	31.70	-0.50	0.22			Shim SR, Lee SM	Jun-24

RS, rivastigmine; MD, mean difference; std, standard deviation; Pla, placebo; SE, standard error; std, standardized mean difference

Confirmation

Those only 4 studies were met our inclusion criteria: (1) studies including patients who had neurodegenerative disorders that can affect cognitive impairments such as Alzheimer's disease (AD) and Parkinson's disease (PD), and/or higher level gait disorders, (2) intervention included prescription of rivastigmine, (3) comparisons were specified as with the inclusion of a control group for evaluating the number of fall or were not specified, focusing solely on the effects of rivastigmine treatment on gait speed, and (4) outcomes were measured as mean differences in gait speed and the number of fall.

The final inclusion of articles was determined through collaborative evaluation meetings involving all authors. In addition, to ensure the integrity of the included studies, the references and collected data were reviewed meticulously so that they did not overlap.
