# Supplementary material for: Effects of rivastigmine on gait in patients with neurodegenerative disorders: A systematic review and meta-analysis
Source: PLoS One. 2024 Dec 12;19(12):e0310900. doi: 10.1371/journal.pone.0310900 (PMC11637393; doi:10.1371/journal.pone.0310900)
Supplement: S5 Table — (PDF) [file pone.0310900.s005.pdf]

Supplementary Table 5. The completed risk of bias and quality/certainty assessments for each study.

a. Risk of bias for RCTs

	D1	D2	D3	D4	D5
Li 2015	Described "randomized" study design	Described "double-blind" study design	The overall dropout rate was 8/89, and the dropout rates between the two groups (Riva stigmine vs. Placebo) were similar.	Both groups (Rivastigmine vs. Placebo) had the same conditions except for drug administration and measured the same outcome (number of falls).	RCT study, results reported according to the research plan
Henderson 2016	Described "randomized" study design	Described "double-blind" study design	The overall dropout rate was 16/130, and the dropout rates between the two groups (Riva vs. Placebo) were similar.	Both groups (Rivastigmine vs. Placebo) had the same conditions except for drug administration and measured the same outcome (number of falls, gait speed).	RCT study, results reported according to the research plan

b. NOS for observational studies

	Representativeness of the exposed cohort	Selection of the non-exposed cohort	Ascertainment of exposure	Outcome of interest not present at start of study	Comparability of cohorts on the basis of the design or analysis	Ascertainment of outcome	Adequacy of duration of follow-up	Adequacy of completeness of follow-up
Gurevich 2014	The exposure cohort (Rivastigmine) is not a representative group of communities; therefore, no stars were given.	The control group (Placebo) is not a representative group of the community; therefore, no stars were given.	The exposure group is the Rivastigmine treatment group.	The control group is the Rivastigmine non-treatment group.	The analysis was conducted as planned, but no overall covariate-adjusted analysis was presented, so no stars were assigned.	The measurement method of Gait speed is clearly described.	It has been observed to occur for a sufficient period of time.	Long-term observations are fully made.
Shimura 2021	The exposure cohort (Rivastigmine) is not a representative group of communities; therefore, no stars were given.	The control group (Placebo) is not a representative group of the community; therefore, no stars were given.	The exposure group is the Rivastigmine treatment group.	The control group is the Rivastigmine non-treatment group.	The analysis was conducted as planned, but no overall covariate-adjusted analysis was presented, so no stars were assigned.	The measurement method of Gait speed is clearly described.	It has been observed to occur for a sufficient period of time.	No stars were assigned due to the lack of outcome measures from long-term observation.
