# Supplementary figures and images for: Effects of rivastigmine on gait in patients with neurodegenerative disorders: A systematic review and meta-analysis
Source: PLoS One. 2024 Dec 12;19(12):e0310900. doi: 10.1371/journal.pone.0310900 (PMC11637393; doi:10.1371/journal.pone.0310900)

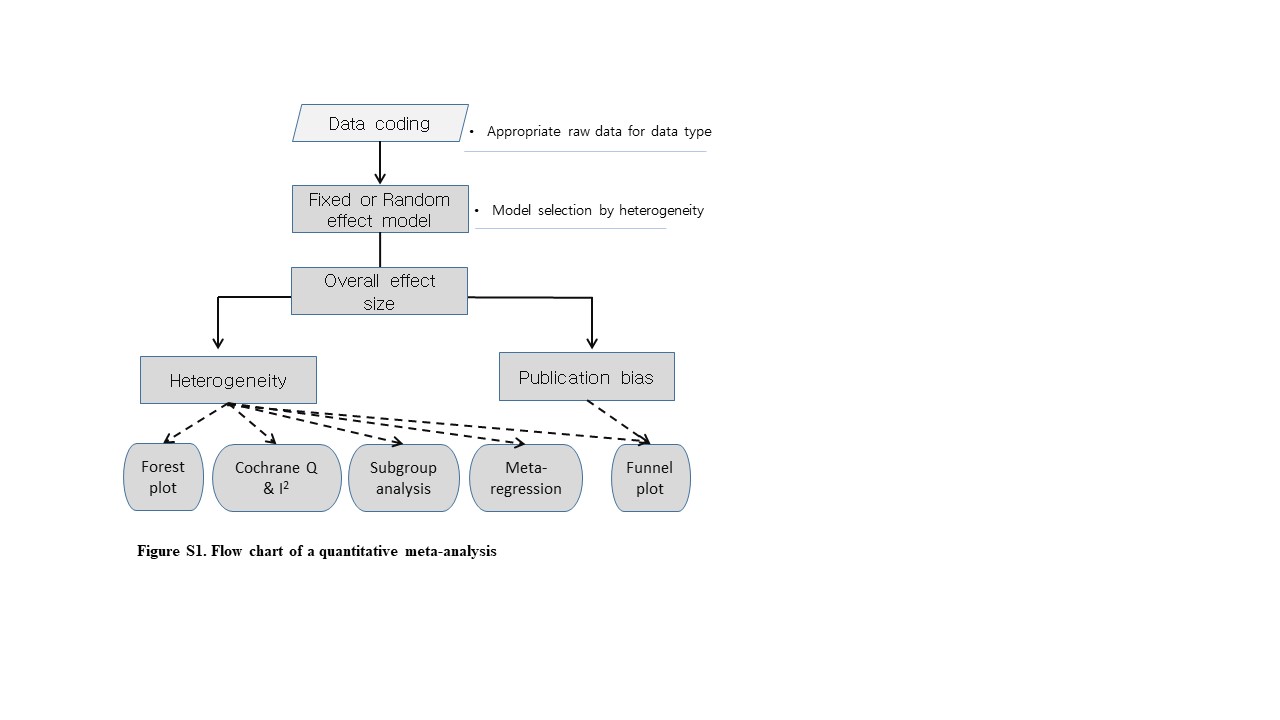

Supplement: S1 Fig — (JPG) [file pone.0310900.s006.jpg]

## Supporting Information

S2 Fig. Funnel plot.

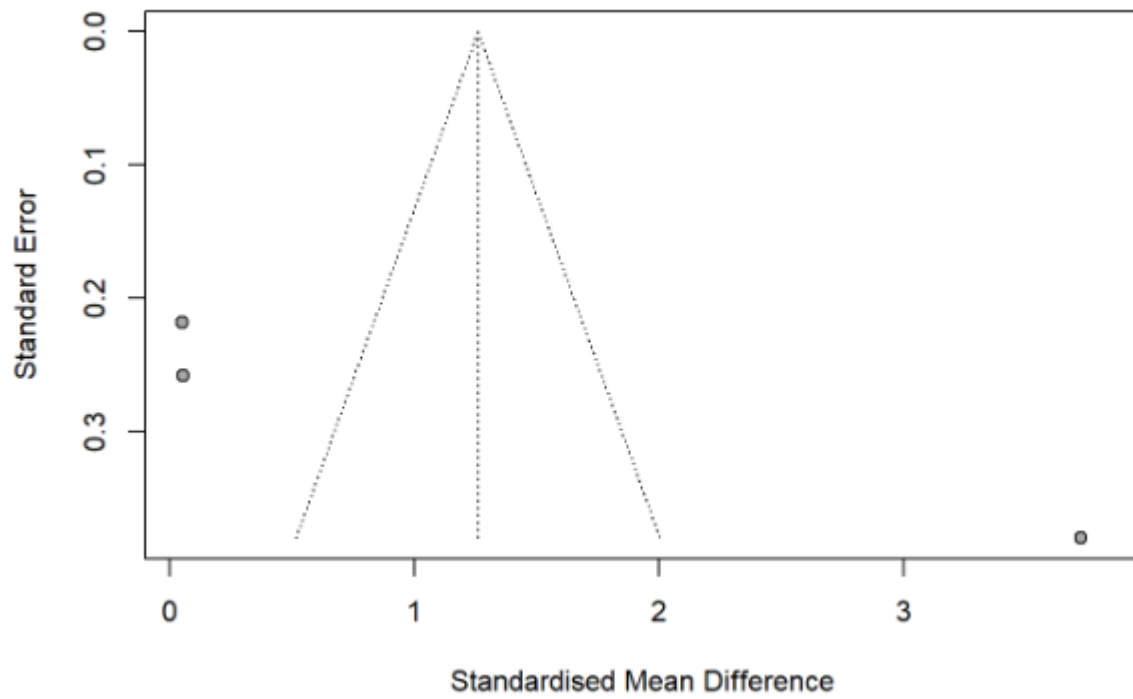

Supplement: S2 Fig — (PDF) [file pone.0310900.s007.pdf]
