# Supplementary material for: Effects of rivastigmine on gait in patients with neurodegenerative disorders: A systematic review and meta-analysis
Source: PLoS One. 2024 Dec 12;19(12):e0310900. doi: 10.1371/journal.pone.0310900 (PMC11637393; doi:10.1371/journal.pone.0310900)
Supplement: Attachment — Submitted filename: ### Response to Reviewers_Neurology 20240802.docx [file pone.0310900.s008.docx]

Dear editor and reviewers of this paper

The authors sincerely appreciate the editorial office and reviewers' efforts to consider this paper for considering publication. We have tracked the changes in the text to show all amendments and responses. Based on the reviewers’ suggestions, we have carefully revised the paper as we can, and include here a point-by-point response to all comments. We marked added sentences, page, and paragraph numbers in blue in the modified file, and specific responses in light blue.

Additionally, in response to the editor's valuable request, we have updated the PRISMA flow diagram and checklist to the new 2020 version. We also revised the total number of articles from 219 to 222, including three articles identified through manual search.

We hope the editorial office and the reviewers like this revised paper. Thank you for considering this manuscript for publication. We look forward to hearing from you.

Sincerely yours,

Seon-Min Lee, MD

Department of Neurology, Konyang University College of Medicine, 158 Gwanjeodong-ro, Seo-gu, Daejeon, 35365, Republic of Korea, Tel.: +82-42-612-2191, Fax: +82-42-600-9090, Email: nestoml@kyuh.ac.kr

**(Reviewer #1:**)

Thanks for your manuscript on this important topic.

I noticed some points in the manuscript that needed to be clarified:

1. In your search strategy, it was better to conduct a literature search in more databases such as Scopus.

**Response:** We really appreciate reviewer 1’s insightful comments. According to your suggestion, we additionally search “Scopus” DB for strict literature searching. At first literature retrieving from DBs of Figure 1, we gathered 222 (PubMed 23, Embase 139, Cochrane 19, and Scopus 38). However, additional 38 studies from Scopus are excluded due to the overlapping data and not related to the research topic as well. We also changed related sentences in Methods and Results section as follows. The PRISMA study selection flow chart in Figure 1 and search queries in S2 Table also have been revised to include the SCOPUS database. Additionally, in response to the editor's request, we have updated the PRISMA flow diagram and checklist to the new 2020 version. *(Figure 1 & search queries in S2 Table: Appendix attached)*

“A total of 222 articles were identified during the initial search across different electronic databases, 50 including PubMed (n = 23), Cochrane (n = 19), Embase (n = 139), Scopus (n = 38), and a manual search (n = 3).” (line 48-50)

"A comprehensive literature search was conducted in the PubMed, MEDLINE, Embase, Cochrane, and Scopus databases" (line 100-101)

“A total of 222 articles were identified during the initial search across different electronic databases, 50 including PubMed (n = 23), Cochrane (n = 19), Embase (n = 139), Scopus (n = 38), and a manual search (n = 3). Of these, 65 studies were excluded because they either contained overlapping data or appeared in multiple databases. After reviewing the titles and abstracts, 130 studies were eliminated because they were unrelated or had trial registrations. Among the remaining 27 full-text articles, 17 were excluded for the following reasons: non-RCTs (n = 17).” (line 171-176)

2. Please provide more data of included studies in table 1.

**Response**: Thank you for your valuable comments. Following your suggestion, we added more information and revised Table 1 to include details such as number of patients, study duration, intervention types, inclusion criteria, and outcome for gait measures.

Each study had diverse inclusion, exclusion criteria, varied methods for gait analysis, different gait parameters, and distinct study designs, resulting in varied results reported in each paper. These diversities made it challenging to compile a consistent table. However, we made every effort to provide as much information as possible. Additionally, in the discussion section, we acknowledged a limitation of this analysis, which was the inclusion of only simple gait analyses. *(Table 1: Appendix attached)*

**(Reviewer #2:**)

The manuscript titled "Effects of Rivastigmine on Gait in Patients with Neurodegenerative Disorders: A Systematic Review and Meta-Analysis" investigates the impact of rivastigmine on gait in individuals with neurodegenerative disorders, such as Alzheimer’s and Parkinson’s disease. This systematic review and meta-analysis assess rivastigmine's effect on gait speed and fall reduction. The comprehensive literature search yielded four key studies with 286 participants. While rivastigmine did not significantly improve gait speed (pooled SMD: 0.761, 95% CI: -1.165 to 2.688), it significantly reduced the number of falls (pooled SMD: -0.366, 95% CI: -0.650 to -0.083). These findings suggest that rivastigmine may help reduce fall risk in patients with neurodegenerative disorders. The manuscript is recommended for publication after my concerns.

**Response**: Thank you for your valuable comments. We tried to make a comprehensive decision on the patient's gait speed and risk for falls according to Rivastigmine treatment.

Here are my concerns;

1. “Fig 2. Forest plots. SMD, standardized mean difference. The random-effects model created using the restricted maximum-likelihood estimator. CI, confidence interval. The black diamond shows the overall effect size. In gait speed, the effect of the rivastigmine single group (pre-post). In fall numbers, the effect of the rivastigmine versus placebo”. The author should explain and add more here.

**Response**: Thank you for your valuable comments. In lines 111-113 of the manuscript, we have already described that gait speed was evaluated using a pre-post group without a control group to assess the effects of rivastigmine, while fall number was compared with a control group. Specifically, the individual studies had discrepancies in parameters, control groups, and methods for measuring gait speed, making it impossible to establish a uniform control group. In response to the reviewer's suggestions, we have added the following explanations in the Results section and the Limitations section.

"The pooled SMD for the overall gait speed from baseline without a comparison group was 1.260 (95% CI: −1.123 to 3.643), indicating no significant improvement in gait speed." (line 191-192)

"Additionally, owing to the differences in gait parameters, controls, and methods of gait assessment employed in each study, our analysis was restricted to a simple gait analysis without control group. " (line 305-306)

2. “Fig 3. Quality assessment. Risk of bias for randomized controlled trials and Newcastle–Ottawa quality scale for observational studies”. The author should explain and add more here.

**Response**: We really appreciate reviewer 2’s insightful comments. According to your suggestion, we added the related sentences of quality assessment in Results section as follows.

“With a risk of bias of 2.0, all studies were rated as having a “low” risk of bias. Li(2015) and Henderson (2016) were recognized as high-quality studies with low bias due to their randomization and blinding methods. Additionally, these studies had obtained prior IRB approval, resulting in minimal risk of missing data and reporting bias. In NOS, all studies were rated with a “Fair” grade. However, Gurevich (2014) and Shimura (2021) were deemed to have higher risk due to inadequate description of cohort representativeness in the exposed and non-exposed groups. Nevertheless, the overall assessment was considered “Fair” (Fig 3).” (line 220-226)

3. The author should add one more line in the last part of the abstract.

**Response:** Thank you for your thorough comments. According to reviewer 2’s suggestion, we have modified and added to the final part of the abstract.

“Rivastigmine treatment in patients with neurodegenerative disorders tend to improve gait speed and significantly reduces fall incidence. Given the limited efficacy of current treatments for gait disturbances and falls, dual-ChEIs like rivastigmine could be a promising therapeutic option.” (line 57-60)

4. The author should add a Schematic figure in the last part of the introduction.

**Response:** We fully agree with the reviewer 2’s valuable opinion. According to your suggestion, we added the schematic figure of quantitative analysis in S1.Fig (line 93) (Figure S1*: Appendix attached)*

5. The conclusion should be modified and add more details.

**Response:** We appreciate Reviewer 2’s thorough comments. Following Reviewer 2’s suggestions, we have modified the conclusion section and added more details.

“Treatment with dual-ChEIs, particularly rivastigmine, tends to improve gait speed and significantly reduce the incidence of falls in patients with neurodegenerative disorders. Considering the limited efficacy of current interventions for gait disturbances and falls, these findings highlight the potential of dual-ChEIs as a promising treatment option. However, limitations include patient heterogeneity, differences in gait measurement methods, and a lack of randomized controlled trials (RCTs) specifically investigating rivastigmine's effects on gait. Future well-designed large-scale RCTs are essential to comprehensively elucidate these effects and to clarify the precise associations and mechanisms in patients with neurodegenerative disorders characterized by high fall risk and gait disturbances.” (line 323-331)

6.The authors need to double-check the whole manuscript to remove grammatical errors/ typos/incomplete sentences and non-relative phrases.

**Response**: Thank you for your suggestion. We have carefully reviewed the entire manuscript to correct grammatical errors, typos, incomplete sentences, and non-relative phrases. Specifically, we have revised several sections to ensure clarity and coherence, and we have also consulted a professional editor (https://www.editage.co.kr/) to improve the overall quality of the text. We believe these changes have significantly enhanced the readability and accuracy of our manuscript.

(**Reviewer #3:)**

My comment on the manuscript entitled “Effects of rivastigmine on gait in patients with neurodegenerative disorders: A systematic review and meta-analysis” are as follows.

**Response:** We appreciate your thorough and detailed comments and questions. We have numbered Reviewer 3’s questions sequentially and have addressed similar questions and suggestions collectively in our responses and revisions. Furthermore, while it would be ideal to include detailed explanations addressing all comments and questions in this paper, we kindly ask for your understanding that such content, which extends beyond the scope and hypotheses of this study, has been limited due to word count constraints. We sincerely hope Reviewer3’s kind understating and satisfaction.

1.What are the primary mechanisms by which cholinergic deficits contribute to gait disturbances in neurodegenerative disorders? / 19. What specific mechanisms underlie the involvement of cholinergic neurons in maintaining gait and preventing falls? / 28. What cognitive contributions to gait and falls have been identified, and what further research is needed to clarify these mechanisms? /14. What future research directions are suggested by the results of this systematic review and meta-analysis?

**Response:** Thank you for the reviewer 3’s essential questions for our study. The cholinergic system, mediated by acetylcholine, plays a crucial role in both the central and peripheral nervous systems. It is involved in regulating motor control, autonomic control, and cognitive processing. Notably, acetylcholine is critical for maintaining alertness and cognitive functions, particularly executive function and attention. These cognitive controls are fundamental mechanisms for ensuring safe mobility, including gait and balance maintenance. Therefore, while cholinergic deficits associated with neurodegenerative disorders can lead to gait disturbances through various mechanisms such as impaired motor control, autonomic regulation, and sensory integration, cognitive decline is a crucial contributing factor. This assertion is supported by various research findings. We have discussed these points in detail in the discussion section of our manuscript. (line 238-255)

Furthermore, while understanding the mechanisms through which cognitive function contributes to gait and falls necessitates multidisciplinary research encompassing both clinical and basic science fields, it is crucial for studies employing meta-analytical methodologies to focus on well-designed, large-scale randomized controlled trials (RCTs). This point has also been addressed in the manuscript. (line 318-320)

2. How was the integrity of the included studies ensured during the review process?

**Response:** Thank you for the reviewer 3’s thorough question. Systematic review (SR) and meta-analysis is one of the methods to find exhaustive answer for specific questions using all possible retrieved researches. Thus, systematic retrieving of literatures and analyzing the overall effect sizes of statistical methods are most important to here. The methodology of SR is detailed in the Materials and Methods, and Results sections of this paper. For instance, SR requires searching at least two databases, but this study used five databases to ensure the integrity of the research. Additionally, at least two independent researchers should search the literature and extract data using the same procedure. In this study, two independent researchers conducted these tasks, and the overall conclusions were reached through consensus among all researchers.

3.What criteria were used to select the final four studies included in the meta-analysis?

**Response:** In response to Reviewer 3's comments on the study selection process, we have provided an explanation under a separate subheading. The inclusion and exclusion criteria are detailed in lines 109 to 123.

"The inclusion criteria were as follows: (1) studies including patients who had neurodegenerative disorders that can affect cognitive impairments such as Alzheimer’s disease (AD) and Parkinson’s disease (PD), and/or higher level gait disorders, (2) intervention included prescription of rivastigmine, (3) comparisons were specified as with the inclusion of a control group for evaluating the number of fall or were not specified, focusing solely on the effects of rivastigmine treatment on gait speed, and (4) outcomes were measured as mean differences in gait speed and the number of fall. To ensure data accuracy and relevance, duplicate publications and publications that did not contain original articles (conference abstracts only, case reports, case series, review articles, editorials, letters, and guidelines) were excluded from the analysis. Two independent investigators (SRS and S-ML) primarily analyzed the titles and abstracts, and then reviewed full-text articles according to the inclusion and exclusion criteria. A data extraction form was used independently by the authors to extract data. The final inclusion of articles was determined through collaborative evaluation meetings involving all authors. In addition, to ensure the integrity of the included studies, the references and collected data were reviewed meticulously so that they did not overlap." (line 109-123)

4.What were the characteristics of the 286 participants across the four studies, and how might these characteristics have influenced the outcomes? / 5.Were there any significant differences in the methodology of the four studies that could have impacted the pooled results for gait speed and falls?

**Response:** Thank you for the reviewer 3’s question. The characteristics and methodologies of individual participants and studies are well-documented in the targeted studies (Table 1). In this study, we conducted additional meta-regression analyses to identify the potential moderating effects of individual participant characteristics. Factors such as the number of participants, age, gender, and country were all found to be covariates influencing the overall effect size. Thus, next research should consider these potential risk factors. More specific explanation about the characteristics of participants is detailed in lines 205-209 and Table 2.

"This study explored the potential moderating roles of specific variables through meta-regression and meta-analysis of variance models. Table 2 presents the results of these analyses. Gait speed was significantly affected by age (P < 0.001) and female sex (P = 0.004). As age and proportion of female participants increased, gait speed decreased significantly. The number of patients (P < 0.001) and country (P < 0.001) also showed statistically significant impact on gait speed" (line 205-209).

10. What were the limitations of the studies included in the systematic review and meta-analysis, and how might these limitations have affected the results?

**Response:** Thank you for the reviewer 3’s valuable question. As this study is a meta-analysis and systematic review, the primary focus is not on the methodologies of the individual studies but on the statistical significance of the overall effect size derived from synthesizing these studies. While detailed methodologies of the individual studies would allow for subgroup analysis, four of the included studies lacked sufficient methodological descriptions, making such analysis infeasible.

Additionally, as discussed in the Discussion section of the manuscript, this systematic review and meta-analysis were not conducted with controlled trials on real-world patients. Consequently, there may be limitations in directly translating these findings into practical clinical applications. Furthermore, given the small sample sizes, future large-scale trials involving real patients are needed to confirm the results of this study. We also described limitations about this systematic review and meta-analysis in Discussion section. (line 300-312)

6. What specific neurodegenerative disorders were represented in the final four studies, and how did the effects of rivastigmine vary across these disorders? / 16. What criteria were used to establish the diagnosis of neurodegenerative disorders in the patients included in the studies?

**Response:** Thank you for the reviewer 3’s detailed questions. The neurodegenerative diseases included in our study are Parkinson's disease (PD), Alzheimer's dementia (AD), and Higher-Level Gait Disorder (HLGD), diagnosed by movement disorders specialists. These conditions were selected based on the research methodology described in lines 99-107, which involved using MeSH terms to search for studies examining the effects of rivastigmine treatment on gait and falls. The identified studies included these diseases. Each study had diverse study designs and different measurement methods for gait and falls, resulting in varied outcomes.

In the study by Gurevich [18], there was no significant improvement in gait speed or stride time variability in HLGD patients after taking rivastigmine. Shimura's study [20] found no significant results in single task gait for AD patients, but there was a significant improvement in gait speed and cadence during dual task gait trials. Henderson's study [5], which was the highest quality study in terms of methodological rigor, focused on PD patients and found a significant improvement in step time variability during normal walking and simple dual task gait, as well as improvements in gait speed and a significant reduction in falls. Similarly, Li's study [19] on PD patients demonstrated a significant reduction in the number of falls when patients took the medication.

As previously mentioned, this study emphasizes the statistical significance of the overall effect size derived from combining the individual studies, thereby providing meaningful insights despite the varied results of each study.

7. How was gait speed measured across the different studies, and were there any inconsistencies in measurement techniques? / 18. How did the researchers ensure the validity and reliability of gait speed measurements across the included studies? /21. What were the differences in gait assessment methods across the studies, and how might these have influenced the results?

**Response:** We appreciate Reviewer 3's important comments on our study. Each study employed different methods for evaluating gait and falls, and the measurement parameters varied. Gurevich et al. [18] used the Timed Up and Go test and measured gait-related parameters with a computerized force-sensitive system. In contrast, Shimura et al. [20] utilized a portable gait rhythmograph equipped with an accelerometer to study various gait protocols. Henderson et al. [5] used walkway system with a triaxial accelerometer and assessed gait during normal walking, simple dual-task walking, and complex task walking, while also measuring the number of falls and administering fall-related scales. Li's study [19] compared annual fall counts and fall incidence.

Therefore, in this meta-analysis, we excluded methods involving cognitive loading, such as dual-task gait and complex-task gait, and focused solely on analyzing gait speed and fall number, which are common parameters measured using simple gait methods. Despite the heterogeneity in methods and measurement indicators across the included studies, statistical techniques will be employed to adjust for these variations. The overall effect size will be derived from combining the individual studies, and statistical significance will be assessed to draw meaningful conclusions. This point has also been addressed in the manuscript. (line 132-137) Additionally, this has been included and addressed as a limitation in the discussion section of the manuscript.

“Additionally, owing to the differences in gait parameters, controls, and methods of gait assessment employed in each study, our analysis was restricted to a simple gait analysis.” (line 305-306)

In addition, meta-analysis is the best way to combine studies based on the same research problem. Therefore, meta-analysis is developing several control techniques for synthesis. For example, heterogeneity was found to be 97% in the gait speed of Figure 2, so a random effect model was adopted to adjust the heterogeneity of individual studies.

8. What potential factors could explain the lack of significant improvement in gait speed despite the reduction in falls?

**Response:** Thank you for the reviewer 3’s detailed questions. As you mentioned, it is indeed an interesting observation. We believe that gait speed and fall risk are distinct attributes influenced by different factors. Specifically, gait speed measures how fast one walks, whereas falls are related to not only gait speed but also the coordination, balance, and stability of various body parts. Moreover, the impact of underlying conditions on gait may vary among individual patients, and the presence of musculoskeletal problems or concurrent medical conditions can also affect detailed gait parameters. Therefore, it is unlikely that a single intervention would lead to improvements in both gait speed and fall risk to the same extent. Additionally, variations in gait measurement methods and protocols across the four studies included in our research may also contribute to this discrepancy.

9. How do the pooled standardized mean differences (SMDs) and Hedges' g compare between the individual studies included in the meta-analysis?

**Response:** Thank you for the reviewer 3’s sharp comments. The SMD is determined by dividing mean difference (MD) by the pooled SD (SMD=MD/SD), and the standard error (SE) equals SD multiplied by the square root of the sum of the inverse of each number of samples. The SMD here is Cohen’s d. If the number of samples is small, the overall effect size tends to be overestimated. To correct this, Hedges’ g is used. Additionally, based on Reviewer 3's insightful comments, we identified and corrected errors in the manuscript as follows.

“The standardized mean difference (SMD, Hedges’g) along with their 95% confidence intervals(CIs)” (line 132-133)

11. How might the duration of rivastigmine treatment have influenced the outcomes observed in the studies?

**Response:** Thank you for the reviewer's comment. As you mentioned, the duration of rivastigmine treatment can have various effects on the outcomes. It is expected that a longer duration of treatment may have a positive impact on gait and falls. However, due to the lack of sufficient treatment duration data, the duration effect could not be confirmed. In fact, there were only four papers in our analysis, which had a great barrier to performing the duration effect. Next study should consider this duration effect as covariates you recommended.

12. Were there any reported adverse effects of rivastigmine treatment in the studies, and how were these managed?

**Response**: Thank you for the reviewer 3’s thorough comment. Each study monitored and managed the side effects associated with rivastigmine treatment appropriately. In the study by Gurevich et al. [18], three patients dropped out due to side effects such as general weakness, fatigue, insomnia, dizziness, nausea, blurred vision, and diarrhea, and two patients discontinued dose escalation. In Shimura et al. [20], there were two cases of fractures unrelated to rivastigmine treatment, and the study continued. However, one patient dropped out due to dermatitis caused by medication intolerance. Henderson et al. [5] reported mainly gastrointestinal troubles such as nausea and vomiting, as well as neurological side effects like dizziness and parkinsonism, but most symptoms were mild and did not require special intervention. On the other hand, Li's study [19] did not report any significant adverse events related to rivastigmine.

*The adverse effects reported in these studies were generally mild, and since they are beyond the scope of this meta-analysis, they were not included in our manuscript.*

13. How do the findings of this meta-analysis compare with previous research on the effects of cholinesterase inhibitors on gait and falls in neurodegenerative disorders? / 27. How do the findings of this study align with previous research on the relationship between acetylcholine activity and fall incidence? / 15. How might individual differences in response to rivastigmine treatment be accounted for in future studies to better understand its effects on gait and falls? / 24. What are the potential benefits of conducting future large-scale RCTs to evaluate the effects of rivastigmine on gait and falls more accurately? 30. What limitations related to patient data and study design should be addressed in future research on rivastigmine and gait improvement? 37. How should future RCTs be designed to effectively measure the impact of dual-ChEIs on gait speed and fall incidence in diverse patient populations?

**Response**: We appreciate reviewer 1’s insightful comments and questions. Numerous clinical and basic researches have investigated the effects of acetylcholinesterase inhibitors(AchEI) on gait in patients with Parkinson’s disease(PD) and Alzheimer’s disease(AD). Additionally, as highlighted in our study, there are four studies included in our analysis and a currently ongoing large-scale RCT (*Ref. 1*) that specifically investigate the effect of the dual cholinesterase inhibitor(DChEI) rivastigmine on gait. Among these, there are two notable meta-analyses that evaluate the efficacy of AchEI in improving gait in PD. (*Ref. 2 and Ref.3*)

*. Ref1. Neumann et al.Cholinesterase inhibitor to prevent falls in Parkinson's disease (CHIEF-PD) trial: a phase 3 randomised, double-blind placebo-controlled trial of rivastigmine to prevent falls in Parkinson's disease. BMC Neurol. 2021; PMID: 34715821*

*. Ref 2. Pagano et al.Cholinesterase inhibitors for Parkinson's disease: a systematic review and meta-analysis. J Neurol Neurosurg Psychiatry. 2015; PMID: 25224676*.

*. Ref3. Chen et al. Cholinesterase inhibitors for gait, balance, and fall in Parkinson disease: a meta-analysis. NPJ Parkinsons Dis. 2021; PMID: 34824258*

In the study by Pangano et al., the treatment of AChEI showed significant cognitive improvements in PD, but did not show a significant reduction in falls. Chen et al.’s study found significant improvement in step or stride variability, but did not observe significant enhancements in gait speed or a reduction in falls. In contrast, our study did not find a significant improvement in gait speed but did observe a significant reduction in falls, which differs from the results of these previous meta-analyses. This discrepancy may be attributed to the inclusion of a broader range of conditions in our study, including PD, AD, and Higher-Level Gait Disorder (HLGD), as well as differences in study design and protocol compared to the studies included in the referenced meta-analyses.

Therefore, it is crucial to ensure a more homogeneous patient population and appropriate control groups with consistent inclusion criteria. Studies should adhere to standardized study and treatment protocols. Additionally, large-scale randomized controlled trials (RCTs) utilizing consistent parameters for cognitive, gait, and fall assessments are needed. Incorporating longitudinal studies alongside these trials would allow for more reliable and thorough monitoring. While conducting such a large-scale RCT with all these requirements may be challenging, it would provide more accurate and dependable results regarding the role of rivastigmine in gait improvement and its benefits. Next study should consider this duration effect as covariates you recommended.

17. How do the effects of rivastigmine on gait speed and falls compare to those of other cholinesterase inhibitors like donepezil and galantamine? / 20. How might the dual inhibition of AChE and BChE by rivastigmine contribute to its effectiveness in reducing fall occurrences? / 22. What role does sustained attention and executive function play in gait regulation, and how does rivastigmine influence these cognitive processes? /31. How does rivastigmine's dual inhibition mechanism enhance acetylcholine levels, and what impact does this have on gait and fall prevention? /36. What are the key differences in the molecular action of dual-ChEIs compared to selective ChEIs, and how do these differences translate into clinical benefits?

**Response**: Thank you for the reviewer's good questions. As discussed earlier, drugs such as donepezil, galantamine, and rivastigmine, which are used to treat dementia, are presumed to have a positive effect on gait by improving cognitive functions, particularly attention and executive function. However, direct evidence supporting this hypothesis is still lacking. Unlike other AChEIs like donepezil and galantamine, rivastigmine inhibits both AChE and BChE.

AChE and BChE are enzymes that break down acetylcholine, a neurotransmitter crucial for cognitive function and motor control. By inhibiting both enzymes, rivastigmine increases acetylcholine levels more comprehensively throughout the brain. AChE is predominantly found at nerve synaptic junctions, while BChE is primarily localized in glial cells. In neurodegenerative diseases such as AD, AChE activity decreases and BChE activity increases as neuronal loss progresses. Rivastigmine addresses this imbalance by inhibiting both enzymes, leading to a more significant increase in acetylcholine levels compared to selective AChE inhibitors like donepezil and galantamine.

Therefore, the objective of this study was to investigate whether this dual inhibition mechanism of rivastigmine would be more effective in improving gait disorders and reducing falls. Our meta-analysis confirmed that while rivastigmine did not significantly improve gait speed, it did show a trend towards improvement, and it significantly reduced the incidence of falls. Although it would have been beneficial to analyze additional gait-related parameters to consider more detailed mechanisms, the absence of common parameters across the studies included in the analysis presented a limitation. We have described these points in the limitations section of the discussion. (line 300-309)

23. How did the study populations' heterogeneity, in terms of different neurodegenerative disorders, impact the overall findings of the meta-analysis? /25. How does the age and sex of patients influence the effects of rivastigmine on gait speed, according to the moderator analysis? / 35. How do variations in patient demographics, such as age and gender, influence the efficacy of dual-ChEIs on gait and fall prevention?

**Response**: Thank you for Reviewer 3's thorough comments. The heterogeneity among study populations, including differences in disease pathology, demographics, individual responses to treatment, and comorbidities, poses challenges for statistical analysis and can affect the reliability and generalizability of the results. However, our study aimed to evaluate the effects of rivastigmine on gait in neurodegenerative disorders with cholinergic deficiency. The impact of this heterogeneity on the results was addressed by integrating individual studies through meta-analytical statistical methods to analyze the overall effect size and statistical significance. Additionally, we included moderator analyses to assess the impact of heterogeneity among study populations. As shown in Table 2, the results of the moderator analysis indicated a tendency for gait speed to decrease with increasing age, and this decrease was slower in females. These findings are consistent with various studies related to gait speed. We have discussed this in detail on lines 206-209 of the manuscript.

"This study explored the potential moderating roles of specific variables through meta-regression and meta-analysis of variance models. Table 2 presents the results of these analyses. Gait speed was significantly affected by age (P < 0.001) and female sex (P = 0.004). As age and proportion of female participants increased, gait speed decreased significantly. The number of patients (P < 0.001) and country (P < 0.001) also showed statistically significant impact on gait speed" (line 205-209).

26. Include some relevant bibliographic studies like PMID: 38784601, PMID: 38774717, PMID: 37489441, PMID: 31996329 & PMID: 27919828 in your manuscript.

**Response**: We appreciate reviewer 3’s detail comments and suggestions. We have thoroughly reviewed the paper recommended by Reviewer 3 and have included relevant content that supports and contributes to the conclusions of our study in the revised manuscript.

“For this reason, there is considerable ongoing research aimed at investigating basic experimental models related to the modulation of the cholinergic system and identifying therapeutic targets in degenerative brain diseases such as AD and PD [30-32].” (line 258-261)

“ Moreover, beyond the currently used donepezil, galantamine, and rivastigmine, research is anticipated on pharmacotherapeutic targets that possess AChE-inhibiting properties and could improve gait and reduce falls in patients with neurodegenerative diseases, thereby enhancing cholinergic function [41, 42].”(line 309-312)

29. How significant is the role of the brainstem cholinergic system compared to the neocortical acetylcholine system in regulating gait and preventing falls? / 32. What specific mechanisms contribute to the observed improvements in gait speed with the use of dual-ChEIs like rivastigmine in patients with neurodegenerative disorders? /33. How do the effects of dual-ChEIs on gait speed and falls compare to those of current standard treatments for gait disturbances and fall prevention?

**Response:** Thank you for the reviewer's comment. Current treatments for gait improvement and fall prevention primarily involve pharmacological approaches, such as increasing the levodopa equivalent dosage. In cases where symptoms like freezing of gait are present, additional medications such as dopamine agonists, MAO-B inhibitors, amantadine, and methylphenidate are often tried. Additionally, some patients may undergo non-invasive brain stimulation techniques, such as Transcranial Magnetic Stimulation (TMS), or surgical treatments like Deep Brain Stimulation (DBS). Recently, there has been a growing emphasis on non-pharmacological approaches, including physiotherapy, sensory cueing, and cognitive training. These methods are recognized for their potential benefits in enhancing gait improvement and preventing falls. However, a standardized treatment protocol for gait and fall has not yet been established. Consequently, there is an increasing focus on the development and validation of effective therapeutic agents for gait and fall-related treatments.

In particular, dual ChEIs are expected to have significant effects and potential for improvement in neurodegenerative diseases accompanied by cholinergic deficits. As Reviewer 3 mentioned, while the role of the neocortical acetylcholine system originating from the basal forebrain in higher-order cognitive functions is important, the role of the brainstem cholinergic system is considered more crucial. The brainstem cholinergic system, particularly the pedunculopontine nucleus (PPN) and laterodorsal tegmental nucleus (LDT), is crucial for posture maintenance, balance, and fundamental gait functions. These brainstem regions project to various parts of the central nervous system, including the thalamus, cerebellum, brainstem, and spinal cord, facilitating the coordination and execution of automated motor activities necessary for stable and continuous walking. They are directly involved in regulating gait patterns and muscle tone required for an upright posture and movement. Therefore, comparing the importance of the two acetylcholine systems depends on the context, and further research is needed to elucidate their precise mechanisms and relationships. Nevertheless, the overall interaction between these systems is likely essential for comprehensive gait regulation and fall prevention. We have addressed the relevant issue in the discussion section (line 238-255).

38. What role do comorbid conditions play in the response of patients with neurodegenerative disorders to dual-ChEIs treatment? /39. How does the severity of cognitive impairment in neurodegenerative disorders affect the outcomes of dual-ChEI treatment on gait and falls?

**Response:** Thank you for the reviewer's thorough questions. As previously mentioned , the key distinction between AChEIs and Dual-ChEIs lies in their action on acetylcholine-degrading enzymes. AChEIs selectively inhibit only AChE, whereas Dual-ChEIs act on both AChE and BChE. This difference suggests that Dual-ChEIs may more effectively maximize ACh levels and, consequently, potentially offer greater benefits in improving gait and reducing falls. Specifically, in AD, it has been experimentally demonstrated that while AChE levels decrease with disease progression, the role of BChE becomes increasingly prominent. Therefore, as cognitive impairment becomes more severe, Dual-ChEIs are expected to contribute more significantly to increasing ACh levels, as discussed in the text (line 262-269) However, comorbid conditions related to ACh can affect the efficacy of Dual-ChEI treatment in various ways. Changes in pharmacological interactions and drug metabolism due to comorbid conditions and concurrent medications can influence the effectiveness of gait and fall improvements. Therefore, it is important to consider interactions with comorbid conditions and related medications when using Dual-ChEIs in patients with neurodegenerative disorders accompanied by cholinergic deficits. *The impact of comorbid conditions and interactions is beyond the scope of this study, and thus, is not included in this paper.*

34. What are the potential side effects and safety concerns associated with long-term use of dual-ChEIs in patients with neurodegenerative disorders? /40. What are the long-term benefits and potential risks of using dual-ChEIs for gait improvement and fall reduction in patients with neurodegenerative disorders? /42. What are the most effective biomarkers for predicting response to dual-ChEI treatment in patients with neurodegenerative disorders?

**Response:** Thank you for the reviewer's valuable questions. Dual-ChEIs commonly cause gastrointestinal side effects such as nausea, vomiting, and diarrhea. They can also induce cardiovascular side effects like bradycardia and hypotension, as well as dizziness due to their anticholinergic effects. Additionally, neuropsychiatric side effects such as insomnia, agitation, and hallucinations may occur, although these are generally not fatal. Concerning safety issues include drug interactions and cholinergic crisis. Therefore, while dual-ChEIs can improve quality of life and increase independence through cognitive benefits, gait improvement, and fall reduction, it is crucial to carefully monitor and manage potential risks to achieve optimal therapeutic outcomes. Effective biomarkers for predicting the response to dual-ChEIs include measuring the activity levels of AChE and BChE in CSF and the plasma ACh level. Clinical and cognitive assessments can also be utilized to predict treatment effects. Combining these various biomarkers can enhance the accuracy of predicting dual-ChEI treatment responses, and it is important to balance side effects and therapeutic benefits appropriately. *Although this is a very important issue, it is beyond the scope of the our study and is not included in this manuscript.*

41. How do environmental and lifestyle factors, such as physical activity levels and home safety, interact with dual-ChEI treatment to influence gait and fall outcomes? / 43. How can patient adherence to dual-ChEI treatment be improved to maximize the therapeutic benefits on gait and fall prevention? / 44. What are the potential implications of dual-ChEI treatment on the overall quality of life and functional independence of patients with neurodegenerative disorders?

**Response:** Thank you to Reviewer 3 for the practical questions. The cognitive and gait improvement effects of dual-ChEI treatment can enhance patients' independent mobility, thereby improving their overall quality of life. Additionally, this may reduce caregiver burden and potentially result in economic benefits. However, it is important to address the safety concerns that may arise from improved mobility by enhancing environmental and lifestyle factors. Maintaining a safe home environment, such as securing loose rugs, modifying slippery floors, and improving lighting, can help reduce fall risks and support patients' independence and mobility. These combined factors can create a synergistic effect, maximizing the therapeutic benefits of dual-ChEI treatment. Improving patients' adherence to medication is also a crucial strategy for maximizing treatment benefits. Simplifying the medication regimen, such as reducing the dosage frequency to once daily using patches or alarms, can make it easier for patients to follow their treatment plan. W*hile these points are significant, they are beyond the scope of this study and have not been included in the manuscript.*

45. How do the costs and benefits of dual-ChEI treatment compare to other therapeutic options for managing gait disturbances and falls? / 46. What additional therapeutic strategies could be combined with dual-ChEIs to enhance their effects on gait and fall prevention in neurodegenerative disorders?

**Response:** Thank you for the reviewer's good comment. As mentioned in response to question 33, in addition to pharmacological treatments such as rivastigmine, various treatment strategies, including non-invasive treatment such as TMS, surgical treatments, physical therapy, and cognitive therapy, can be used to improve gait and prevent falls. Compared to other therapeutic options for managing gait disturbances and falls, dual-ChEI treatment may offer a more favorable balance of costs and benefits. Although the initial cost of the medication may be higher than some non-pharmacological interventions, the significant improvements in cognitive and motor functions can lead to reduced healthcare costs associated with fall-related injuries and hospitalizations. Furthermore, the combined benefits of dual-ChEIs on cognitive and motor functions provide superior outcomes compared to treatments that target only motor symptoms or cognitive decline. However, determining the best combination of treatments and the most cost-effective approach requires consideration of individual patient needs and the overall healthcare context. *Although these considerations are important, they are beyond the scope of this study and have not been included in this manuscript.*
